# Clinical Heinz Body Anemia in a Cat After Repeat Propofol Administration Case Report

**DOI:** 10.3389/fvets.2020.591556

**Published:** 2020-10-26

**Authors:** Courtney L. Baetge, Lauren C. Smith, Carolina P. Azevedo

**Affiliations:** Department of Small Animal Clinical Sciences, Texas A&M University, College Station, TX, United States

**Keywords:** Heinz body, anemia, propofol, feline, anesthesia, repeated, case report

## Abstract

Heinz body formation has been reported in cats repeatedly administered propofol for anesthesia induction, although the resultant changes were deemed of little clinical significance (1, 2). This report suggests repeated propofol administration to some individual cats might induce anemia with clinical signs and cessation of propofol administration may result in rapid resolution. A 9-years-old American Domestic Shorthair cat receiving a 20-fraction radiation protocol for lateral thoracic fibrosarcoma showed lethargy, decreased appetite and activity, and Heinz body (3+ on blood smear examination) anemia (packed cell volume 22%; reference interval 24–45%) after 12 repeated propofol anesthesia inductions. The anesthesia induction protocol was adjusted to exclude propofol. Over the following week, the anemia resolved (packed cell volume, 30%), and the cat's activity level, appetite and attitude improved. The total dose of propofol received over the 12 treatments was 62.4 mg/kg.

## Introduction

This case report describes a 9-years-old castrated male 6.2 kg American Domestic Shorthair cat that developed Heinz body anemia and showed clinical signs of lethargy and decreased appetite and activity after 12 propofol anesthesia inductions. The cat was presented to the Texas A&M College of Veterinary Medicine & Biomedical Sciences for radiation treatment of a previously diagnosed fibrosarcoma and was found to have a cardiac murmur and several bloodwork abnormalities. After 12 propofol anesthesia inductions the cat became depressed, lethargic and anorexic and hematology revealed anemia (packed cell volume [PCV] 22%; reference interval [RI] 24–45%; hemoglobin 7.6 g/dL; RI 8.0–15.0 g/dL) with 25–50% of the red blood cells containing Heinz bodies. After alteration of the anesthesia induction protocol excluding propofol, the cat began to show clinical improvement within 3–5 days. One week after discontinuing propofol, PCV was 30% and clinical signs had resolved. Previous publications have noted Heinz body formation in cats after repeated propofol inductions but have concluded this was clinically irrelevant and none have provided follow-up regarding resolution of the Heinz bodies (1, 2). Propofol is one of the most commonly used anesthesia induction drugs for repeated anesthesia due to its smooth induction and quick recovery. This case report shows that in some individual cats, repeated use of propofol for anesthesia induction may lead to Heinz body anemia with clinically relevant symptoms and should be a rule-out in these situations. It also provides a timeline for clinical recovery after cessation of propofol for anesthesia inductions.

## Case Description

A 9-years-old castrated male 6.2 kg American Domestic Shorthair cat was presented to the Texas A&M College of Veterinary Medicine & Biomedical Sciences for radiation treatment of a previously diagnosed fibrosarcoma on the left lateral thorax. The tumor was excised 2 months previously, and histopathologic examination determined it to be an intermediate-grade fibrosarcoma. One month after surgery, a subcutaneous nodule under the thoracic, healed, surgical incision scar was noted and presumed to be recurrence of the tumor.

On presentation, the patient was bright, alert, and cooperative with a grade 2/6 parasternal systolic cardiac murmur, healed 5 cm scar, and a 1 cm subcutaneous firm nodule at the caudal end of the scar. Complete blood count (CBC) showed a packed cell volume (PCV) of 38% (reference interval [RI] 24–45%), thrombocytopenia (platelets, 48,000/μL; RI 300,000–800,000; 2+ clumping], lymphopenia (lymphocytes, 645/μL; RI 1,500–7,000), and blood smear showed mild poikilocytosis, anisocytosis, keratocytosis, thrombocytopenia and marked (3+) echinocytosis (Table 1). Serum chemistry abnormalities included hypophosphatemia (phosphorus, 3.0 mg/dL; RI, 3.8–7.5) and hyperlactatemia (lactate, 35.8 mg/dL; RI, 5.4–15.3). CBC and serum chemistry were evaluated by the clinical pathology laboratory at Texas A&M College of Veterinary Medicine & Biomedical Sciences and read out by a boarded clinical pathologist. After the tumor excision by the referring clinic, the patient developed severe post-operative hemorrhage likely due to a slipped vessel ligation that necessitated hospitalization and a blood transfusion. The CBC and serum chemistry abnormalities were evaluated in light of this prior event. The moderate thrombocytopenia was attributed to consumption from this recent acute post-operative hemorrhage and the 2+ clumping. This was confirmed as the platelets continued to trend upwards over the coming weeks. The lymphopenia was mild and considered related to a stress response as the cat was resistant to restraint and blood collection (3). The phosphate value was low for our lab but considered within range for some references (3). Hypophosphatemia can be due to an increased urinary loss, decreased absorption or transcellular shift. Transcellular shift can be seen with a change in serum pH (diabetic ketoacidosis, respiratory alkalosis) and insulin administration. The mostly likely causes in this cat are respiratory alkalosis as the cat was resistant to restraint and blood collection or malabsorption due to his geriatric age. Mild hypophosphatemia without hypercalcemia is considered of little clinical importance (3). Hyperlactatemia is seen mainly with tissue hypoperfusion but can also be seen in cephalic samples, blood samples with delay in processing or in patients that struggle significantly during or just prior to blood sampling (3). Studies have shown as much as a 4-fold increase in cats subjected to a 5-min struggle with a mean lactatemia of 64 mg/dL (4). This cat was fairly resistant and had a physical exam prior to the blood draw. No signs of hypoperfusion were noted on the physical exam. Echocardiography by a boarded veterinary cardiologist showed focal thickening at the basilar septum, which was interpreted, based on the normal left atrial size, as an early manifestation of hypertrophic obstructive cardiomyopathy. Oncologic treatment options were discussed and radiation therapy followed by wide surgical excision was elected. A protocol of 20 daily fractions (Monday through Friday for 4 weeks) with a helical TomoTherapy unit (TomoTherapy Inc., Madison, WI) was prescribed. The cat would return home with owners each evening after treatment and during the weekends.

**Table 1:** Hematologic changes seen in a 9 years old cat during repeated anesthesia with propofol.

	**Week of treatment**							**Reference interval**
	**Presentation**	**Week 1**	**Week 2**	**Week 3**	**Week 4**	**Week 5**	**2 weeks post-txt**	
PCV	36	43	37	36	22	30	38	24.0–45.0%
WBC	4.3	5.6	4.2		3.4	3.5	4.1	5.5–19.5 × 10^3^/μl
RBC	8.03	9.54	8.42		5.11	5.66	7.92	5.00–10.00 × 10^3^/μl
HGB	12.2	14.2	12.8		7.6	9.3	12.3	8.0–15.0 g/dl
MCV	47.9	46.6	45.2		46.7	51.7	50.4	39.0–55.0 fl.
MCHC	31.8	32	33.6		31.7	31.8	30.7	31.0–35.0 g/dl
PP	6.8	7.4	7.3	6.9	6.7	6.6	7.3	6–8 g/dl
PLT	48,000	197,000	150,000		360,000	375,000	248,000	300,000–800,000/μl
NEUT	3,354	4,704	3,066		2,652	2,975	3,116	2,500–12,500/μl
LYMPH	645	336	588		408	280	451	1,500–7,000/μl
MONO	172	168	252		136	105	82	0–850/μl
EOS	129	392	294		204	140	451	0–1,500/μl
ABS RETIC	n/a	n/a	n/a		n/a	30,100	n/a	<60,000/μl
PlT EST	Low w/2+ clumps	Low	Clump		Clump	Adeq	sl low	
NEUT MORPH	Normal	Dohle bodies	Normal		Dohle bodies	Dohle bodies	Normal	
RBC MORPH	Sl anisocytosis 1+ poikilocytosis 1+ keratocytes 3+ echinocytes	Sl anisocytosis Rare acanthocytes 2+ echinocytes	Sl anisocytosis 1+ echinocytes		Sl anisocytosis Sl rouleaux 1+ poikilocytosis 3+ Heinz bodies 2+ echinocytes	Mod anisocytosis 1+ echinocytes	Sl rouleaux Sl anisocytosis	

*Rare = 1 per 2–3 high power field, 1+ = 1–10%, 2+ = 11–25%, 3+ = 26–50%, 4+ = >50%; PCV, packed cell volume; WBC, white blood cell count; RBC, red blood cell count; HGB, hemoglobin; MCV, mean corpuscular volume; MCHC, mean corpuscular hemoglobin concentration; PP, plasma protein; PLT, platelet count; NEUT, neutrophil count; LYMPH, lymphocyte count; MONO, monocyte count; EOS, eosinophil count; ABS RETIC, absolute reticulocyte count; PLT EST, platelet estimate; NEUT MORPH, neutrophil morphology; RBC MORPH, red blood cell morphology; The reference intervals are lab specific and fit 95% of the values from healthy animals*.

For CT imaging and radiation planning, the cat was pre-medicated with methadone (0.2 mg/kg IV) and general anesthesia was induced with alfaxalone (1.3 mg/kg) and midazolam (0.2 mg/kg IV). The cat was intubated and anesthesia was maintained with sevoflurane in 100% oxygen. The anesthesia was uneventful, but upon recovery, he became very dysphoric, forcefully tried to remove the IV catheter bandage and was aggressive when touched, for 30–45 min. Despite changing premedication to butorphanol then dexmedetomidine, the cat continued to show unacceptable recoveries after anesthesia. Therefore, it was decided to exclude alfaxalone from the anesthesia induction protocol.

For the fourth anesthetic event, the cat was premedicated with dexmedetomidine (2 mcg/kg IV), and anesthesia was induced to effect with propofol (Propoflo® 10 mg/ml; 4.3 mg/kg IV). The cat was intubated and anesthesia was maintained with sevoflurane in 100% oxygen. Recovery was uneventful and this protocol was repeated for the following 11 treatments. Propofol dose ranged from 3.2 to 6.4 mg/kg with a total dose of 387 mg (62.4 mg/kg) used for anesthesia induction over the 12 treatments. Average time for anesthesia maintenance with sevoflurane inhalant was 32 min. At the end of week 3, prednisolone (0.2 mg/kg q24 h PO, and tapered within 7 days to q48 h) was prescribed as an anti-inflammatory due to owner concerns for a slightly decreased appetite and mild inflammation at radiation site.

**Figure 1:**
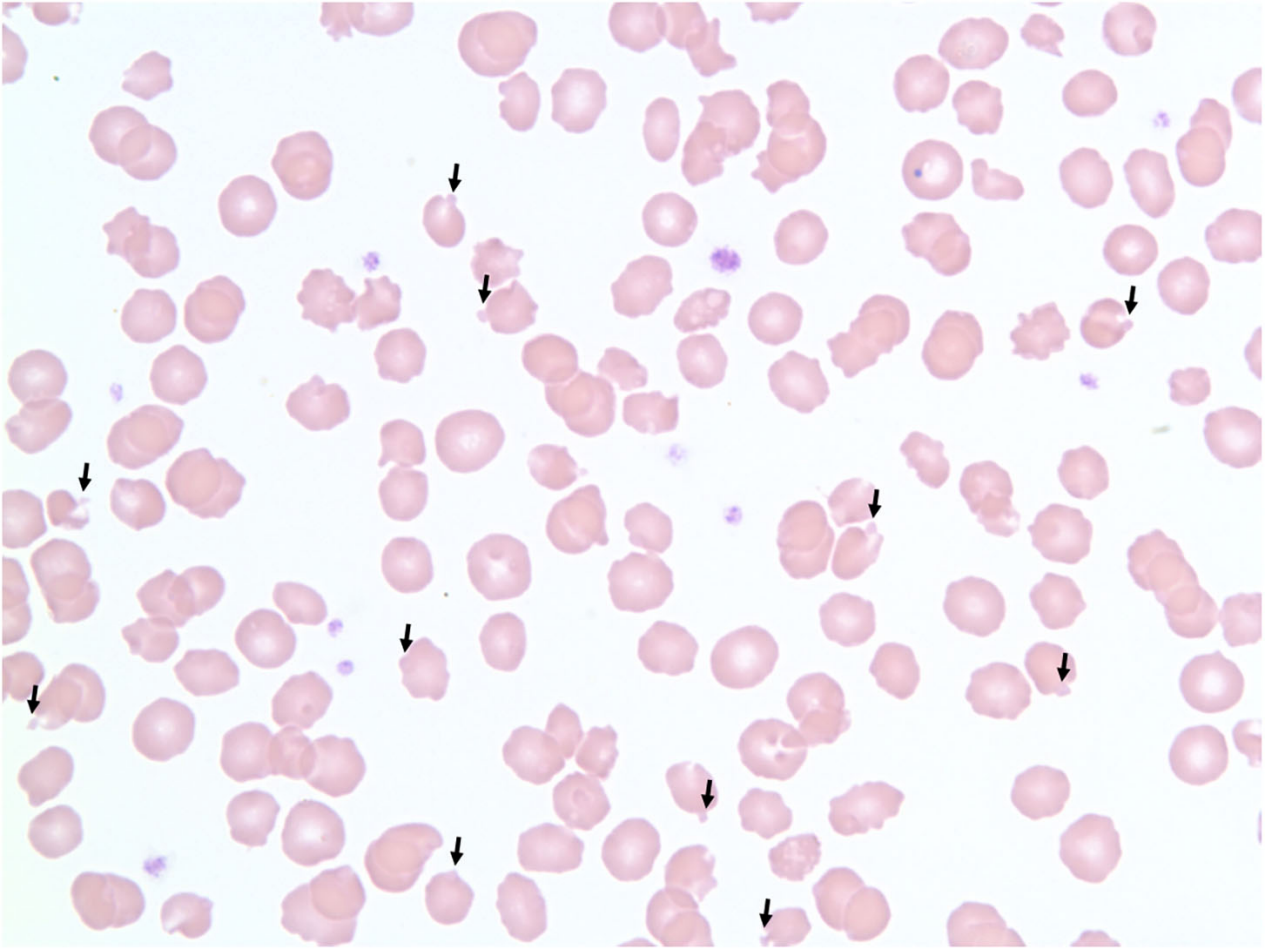
100× magnification blood smear showing red blood cells with Heinz bodies in a cat after repeat propofol administration. Heinz bodies indicated with arrows.

Packed cell volume during the first 3 weeks of treatment had ranged between 36 and 43% (Table 1). Serum chemistry abnormalities included hyperlactatemia that ranged from 24.5 to 27.7 mg/dL (RI 5.4–15.3 mg/dL) and increased ALT activity that ranged from 54 to 101 U/L (RI 26–84 U/L). The cat maintained a normal activity level and appetite at home until the end of week 3 when appetite began to decrease. Physical exam typically showed a bright and alert cat that was moderately resistant and vocal to handling. At presentation on Monday of week-4, however, owners reported significant lethargy and significantly decreased appetite over the weekend. Physical exam noted a quiet demeanor and decreased activity level. The CBC revealed a packed cell volume of 22%, and hemoglobin concentration of 7.6 g/dL (RI, 8–15 g/dL). Blood smear microscopy showed ~25–50% of RBCs contained Heinz bodies (3+) with poikilocytosis (1+), slight rouleaux, slight anisocytosis, and echinocytes (2+) (Figure 1). Serum chemistry was unremarkable with a lactate of 11.5 mg/dL and ALT of 80 U/L. The presence of anemia and Heinz bodies was suspected to be the result of repeated propofol administrations over the previous 3 weeks and anesthesia was canceled for that day.

Packed cell volume the following day had not declined further (23%) so radiation treatments resumed but the anesthetic protocol was changed to midazolam (0.2 mg/kg IV) and ketamine titrated to effect (6 mg/kg IV) for anesthesia induction. The recovery, however, was unsatisfactory, with severe ataxia, anorexia and long-lasting sedation. Due to the high dose of ketamine required for intubation, poor recovery and concerns for HCM in this cat, the anesthetic protocol was modified again for the remaining treatments. Dexmedetomidine (1 mcg/kg IV) was given for sedation, followed by sevoflurane chamber induction then intubation and maintenance on Sevoflurane in oxygen. Owners noted a mild improvement in activity and appetite starting on day 3 of week 4 (5 days after last dose of propofol) and continued improvement through the weekend.

Due to the skipped radiation treatment, treatment spanned into a 5th week. The packed cell volume on day1 of week-5 was 30% with 30,100 reticulocytes. Blood smear showed moderate anisocytosis and echinocytes (1+) with no mention of Heinz bodies. Serum chemistry was unremarkable with a lactate of 11.5 mg/dL and slightly increased ALT activity (136 U/L). The cat showed return of normal appetite and activity per owners over the weekend. Physical exam noted a bright, alert cat that showed some resistance and vocalization with handling once again.

At a recheck 2 weeks after the last radiation treatment, the packed cell volume was 38% (Table 1) with slight rouleaux, slight anisocytosis, <0.50% reticulocytes and no mention of Heinz bodies. Serum chemistry abnormalities included hyperlactatemia (29 mg/dL) and increased ALT activity (88 U/L).

## Discussion

Heinz bodies are inclusions which form within erythrocytes following oxidative damage to the globin portion of the hemoglobin molecule. The oxidative damage results in denaturation and precipitation of hemoglobin, with subsequent binding of the precipitated molecules to the internal surface of the erythrocyte membrane. Heinz body development may result in premature phagocytosis of affected erythrocytes, with a consequent hemolytic anemia. Feline red blood cells (RBCs) have relatively high concentrations of oxidizable sulfhydryl groups and so they are especially prone to Heinz body formation in association with oxidative damage. With a relative deficiency of glucuronide conjugation, they also are less able to defend against oxidative damage. Lastly, the feline non-sinusoidal spleen does not filter out Heinz body red blood cells efficiently (5). Causes of oxidation and Heinz body formation in cats can include diabetes mellitus, lymphoma, hyperthyroidism, genetic enzyme disorders, toxins, drugs, and mineral deficiency. Due to the Heinz body anemia formation after repeated anesthesia and the lack of diagnostics supporting lymphoma, diabetes or hyperthyroidism; we hypothesized the anemia in this cat was due to the repeated propofol administration. Heinz Body formation is a well-recognized finding after repeated propofol inductions in cats (1, 2, 6).

Several studies have been published that examine the effect of repeat anesthesia induction with propofol or induction and a CRI of propofol in cats. Most studies have stated that the repeated induction of anesthesia with propofol in cats may cause slight Heinz body production but no clinical signs and therefore is of little clinical significance. However, the study that included a CRI stopped the study early due to the significant clinical signs. The difference in these study results may be related to the total dose the cats received over the duration of the study.

The study by Matthews which used a dose of 10 mg/kg of propofol on 3 consecutive days in 10 cats and saw a slight increase in number of Heinz bodies to 1+ (<5%) but considered this clinically insignificant (6). In a study by Bley, 13 cats were induced with 6.3 mg/kg of propofol daily for 5 consecutive days. This first group did not show any clinically significant decrease in hematocrit or hemoglobin concentrations. A second group of 24 cats were induced and with 4.7 mg/kg of propofol 12 times over 19 days for a total of 56.4 mg/kg. This second group did show a statistically significant decrease in these parameters after treatment 6 and 12 from baseline. There were also low numbers of Heinz bodies identified in both groups. However, the authors again concluded that “repeated propofol-associated short-duration anesthesia does not lead to clinically relevant hematologic changes in cats undergoing short-duration radiotherapy” (2).

However, in a study by Andress, six cats received propofol for induction plus a CRI of propofol. The cats received repeated induction doses (6 mg/kg IV) as well as a CRI of propofol (0.2–0.3 mg/kg/min for 30 min) for up to 7 consecutive days. This study found no hemolysis or anemia in any cat but did see increased recovery time by ~25 min after day 2, clinical signs of malaise, anorexia, and/or diarrhea on days 5, 6, and/or 7, increased Heinz body formation up to 22–31% on day 7, and facial edema in 2 cats (1). Andress comments in his study that Heinz body formation increased significantly on Day 4 which correlated with a total dose of 40–60 mg/kg similar to what is seen in the cat in this case report. Although hemolytic anemia was not detected in this study, the red blood cell parameters were not measured after cessation of propofol. A decrease in red blood cell lifespan secondary to Heinz body formation with resultant regenerative anemia may not have been detected for several days after propofol cessation. None of the above studies followed the cats after cessation of propofol use to determine timeline for resolution of the Heinz bodies.

Although causation cannot be proven, this case report suggests that in certain cats, the Heinz body formation associated with repeated propofol administration can become clinically relevant and induce anemia with associated clinical signs. This may be related to the duration and total dose of propofol the cat receives and may explain the variation in Heinz body formation and lack of anemia seen in the previous studies (1, 2, 6). Matthews study was over only 3 day with a total dose of 30 mg/kg and saw minimal hematologic changes. In the Bley study, the first group had a lower dose but longer duration (6.3 mg/kg and 5 days, respectively) equaling a similar total dose of 31.5 mg/kg and also saw minimal changes. However, the second group had a duration of 12 treatments over 19 days with a total dose of 56.4 mg/kg. With this increased dose and duration, the cats demonstrated statistically significant changes in hematologic values but none that were deemed clinically significant by the author. However, the Andress study saw such significant clinical changes within 4 days when the total dose reached 40–60 mg/kg, that the study was stopped prior to the planned 10-days duration due to the severity of these clinical signs. These studies support the idea that the total accumulated dose as well as the duration of propofol administration maybe the significant factors when considering Heinz Body formation due to propofol administration. None of these studies had long term follow-up to look for anemia or resolution of the Heinz bodies. No other major changes were made in the cat in this case report during this period and radiation treatment was continued with only a 1-day delay, so we believe that the improvements in hematologic and clinical status are directly associated with the cessation of propofol administration. We also report that within 4 days of the last propofol administration there was an improvement in clinical signs and full resolution within 1 week of cessation of propofol. Thus, Heinz body anemia due to repeated propofol administration may be clinically relevant and may require a change in protocol in certain cats. A total dose over the duration of the treatment exceeding 40 mg/kg maybe a factor contributing to the Heinz body formation. Also, cessation of propofol administration can resolve the Heinz body anemia within as short of a time period as 4–7 days.

## Data Availability Statement

The original contributions presented in the study are included in the article/supplementary material, further inquiries can be directed to the corresponding author/s.

## Ethics Statement

Consent was obtained from the owners prior to the writing and submission of this case report.

## Author Contributions

CB assisted in case management and wrote the manuscript. LS was in charge of primary clinical management of the case. CA was involved in the clinical pathology of the case and wrote sections of the manuscript. All authors critically reviewed and approved the final version of the manuscript.

## Conflict of Interest

The authors declare that the research was conducted in the absence of any commercial or financial relationships that could be construed as a potential conflict of interest.
